# Uptake and Binding of At‐211 Into K‐ and Cs‐Derivatives of Alpha‐Zirconium Phosphate Nanoplatelets for Use as a Targeted Alpha Therapy Delivery Platform

**DOI:** 10.1002/smsc.202500640

**Published:** 2026-04-18

**Authors:** B. D. Imansha Madhushan, Adrianna L. Orsi, Jehan S. Perera, Jennifer M. Pyles, Christine C. Lawrence, Marcus Le, Gabriel C. Tabacaru, Shayden R. Fritz, Lauren A. McIntosh, Sherry J. Yennello, Jonathan D. Burns

**Affiliations:** ^1^ Department of Chemistry University of Alabama at Birmingham Birmingham Alabama USA; ^2^ Cyclotron Institute Texas A&M University College Station Texas USA; ^3^ Department of Chemistry Texas A&M University College Station Texas USA

**Keywords:** astatine‐211, targeted alpha therapy, zirconium phosphate

## Abstract

The ion exchange behavior of K‐ and Cs‐derivatives of α‐zirconium phosphate, A‐ZrP, with the targeted alpha therapy (TAT) radionuclide ^211^At, as At^+^ and AtO^+^, has been investigated. The K‐ZrP shows strong affinity for both At^+^ and AtO^+^, ≥99% uptake. The affinity to Cs‐ZrP was less pronounced, 87%–94% uptake, favoring At^+^. The binding strength was tested against several leaching solutions, including carbonate, phosphate buffered saline (PBS), 4‐(2‐hydroxyethyl)‐1‐piperazineethanesulfonic acid (HEPES) buffers, and ethylenediaminetetraacetic acid (EDTA) solutions at various concentrations (0.1–10 mM). K‐ZrP retained ^211^At in all buffer and EDTA solutions up to 1 mM (<0.5% leaching). The Cs‐ZrP showed no leaching of At^+^, while AtO^+^ leached (1%–3%) in the carbonate and HEPES buffers, along with all of the EDTA solutions, with complete retention only in the PBS buffer. In all cases, when the EDTA concentration reached 10 mM, ^211^At leaching was observed. Once incorporated into the ZrP nanoplatelets, significant shielding of the α‐particles was observed, not only attenuating the intensity of the emission but also reducing the energy of the α‐particles themselves exiting the nanoplatelets. These properties provide the basis for K‐ZrP, and to a lesser extent, Cs‐ZrP to be further considered as potentially promising candidates for a delivery mechanism of ^211^At for application in TAT.

## Graphical abstract

Intercalation of At‐211 between the layers of K‐ or Cs‐zirconium phosphate.

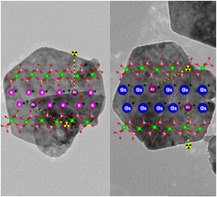

## 1 Introduction

The rapidly growing field of targeted alpha therapy (TAT) for the treatment of various kinds of cancer has attracted much attention recently [1, 2, 3]. The TAT drugs link α‐particle‐emitting radionuclides to tumor‐selective carrier molecules. These tumor‐selective molecules are considered to be the delivery mechanism or vehicle that transports the α‐emitter to the tumor site. This allows the high linear energy transfer of the α‐particles themselves to be centered on the cancerous cells, and the resultant short path length in human tissue, <100 µm, allows a very high cytotoxic radiation dose to be delivered to the targeted tumor of interest, while minimizing damage to surrounding healthy cells [4]. This is a distinct advantage over other types of radionuclides that emit beta‐ or gamma‐radiation, which penetrate much further into healthy tissue. One of the most promising TAT radionuclides is ^211^At, among others [4, 5].

One of the largest challenges associated with ^211^At is the knowledge gaps that exist for At in general [6, 7], which is most certainly a result of it being the lowest abundant element in the Earth's crust, estimated at 0.07 g [8]. Of the known isotopes of At, the longest lived is ^210^At, with an 8.1 h half‐life, followed by ^211^At, with a 7.2 h half‐life. This produces logistical challenges to studying At, in that any experiment performed has to be in relatively close proximity to the production facility. The production of ^211^At is generally conducted at a particle accelerator capable of accelerating α‐particles between 28.5 and 31 MeV to facilitate the ^209^Bi(α,*x*n)^213‐*x*^At nuclear reaction [9, 10, 11, 12]. The number of facilities that are able to produce such a beam is growing; however, the last assessment in 2021 identified roughly only 20 sites worldwide, with just 7 of those being in the United States [13]. Despite these hurdles, there is a rapidly growing interest in investigating the utility of a class of TAT drugs based on ^211^At [4, 14, 15, 16, 17, 18, 19, 20, 21, 22, 23], leading to several clinical trials [24, 25, 26, 27, 28, 29, 30, 31, 32, 33].

To enable expansion in the use of ^211^At, our group has been focused on filling some of the knowledge gaps that exist and providing solutions to some of the problems involving the fundamental chemistry and widespread availability of ^211^At. A large amount of our effort has centered on growing the basic understanding At chemical properties, by examining its distribution across a variety of phase boundaries, including liquid–solid [34, 35] and liquid–liquid [36, 37, 38, 39] extraction systems. From this, a unique interaction of AtO^+^ with ketone functional groups was discovered [36, 37, 38, 40]. This discovery led to a separation system, which could rapidly separate, purify, and recover ^211^At from the Bi target, along with any other radiochemical contaminants produced during the bombardment [41, 42, 43], while providing a means to produce shippable column cartages capable of housing 10s of mCi of ^211^At [44]. The ^211^At‐loaded cartridges have been shipped from Texas A&M University to both the University of Alabama at Birmingham and the Texas MD Anderson Cancer Center, where follow‐on reactions have been performed [41, 45, 46, 47]. These studies have demonstrated that this approach can serve to significantly increase the availability of ^211^At outside of the production facilities.

Once produced and delivered to a facility, the problem of formulating the ^211^At into a radiopharmaceutical also presents several challenges. Administering the cytotoxic payload to the tumor or cancerous cells, while minimizing its effects on surrounding healthy cells, is a challenge that all drug delivery platforms face. The targeting of the diseased cells requires a biochemical tag, which is highly specific; many different species have been investigated, including antibodies [16], peptides [48], proteins [13], or other organic molecules [49]. In the case of TAT drugs, the delivery platform must also be resistant to damage from the emission of α‐particles and the subsequent recoil of the daughter nuclei, resulting in a tremendous amount of damage to the local environment, known as radiolysis. This limits the use of traditional labeling approaches that employ organic ligands vulnerable to degradation, and is further complicated by the fact that the carbon–halogen bond weakens going down the column, which has highlighted the challenge of the so‐called in vivo deastatination [50, 51, 52, 53]. Inorganic materials could provide a solution to this problem; particularly, nanomaterials have attracted interest in this area [54]. Several classes of materials have been investigated with some measure of success, ranging from polymer‐based nanoparticles [55], lanthanum vanadate nanoparticles [56, 57], lanthanum phosphate nanoparticles [58], and carbon nanotubes [59]. Specifically, for ^211^At, silver and gold nanoparticles have received attention, taking advantage of their tunable size and shape, along with their soft metal character. Successful conjugation with both silver and gold nanoparticles for targeted drug delivery applications in several studies has been achieved [60, 61, 62, 63, 64, 65]. However, in these cases, the conjugation is accomplished by organic surface ligands, which face similar challenges to those of traditional labeling pathways.

The alpha phase of zirconium phosphate (α‐ZrP) is an acidic material, which has two exchangeable protons, Zr(HPO_4_)_2_·H_2_O, so that it can participate in ion exchange reactions [66, 67]. The ion exchange properties of α‐ZrP, which have been of primary focus since its discovery by Clearfield and Stynes in 1964 [66] and the following decades [68, 69, 70, 71, 72, 73, 74], contribute to its potential as a TAT vehicle candidate [72, 75, 76]. There are several advantages α‐ZrP offer as a TAT vehicle, including (1) no competing metabolic function and minimal or no cytotoxicity [77, 78], (2) stability under biological conditions [79], (3) extremely high synthetic purity (>99.99%) [67], (4) known crystal structure [70, 80], (5) a unique nano‐platelet shape [67, 79], (6) chemically modifiable surface [81, 82, 83, 84, 85, 86], and (7) tunable particle size [82, 87]. In particular, the nanoplatelet shape is amenable to the enhanced permeability and retention (EPR) effect, which may provide better adhesion, margination, and binding properties over symmetrically shaped nanomaterials. The accumulation of the ^211^At‐loaded‐ZrP nanoplatelets at the diseased site will allow the emission of α‐particles to centralize around the tumor to inflict a lethal radiation dose without the need for cell internalization. All this taken together, the chemical properties (i.e., ion‐exchange intercalation chemistry and the ability to functionalize the surface), along with the physical properties (i.e., particle shape and size), make α‐ZrP very attractive to investigate for radionuclide delivery.

Investigations into using ZrP as a drug delivery mechanism have previously explored intercalating things like cisplatin (cisPt) [88], doxorubicin (DOX) [79, 84], and insulin [77]. While we have focused on the intercalation of metal cations relevant to radiopharmaceutical applications, specifically, La^3+^, Cs^+^, and Bi^3+^, to mimic members of the ^225^Ac decay chain, ^225^Ac^3+^, ^221^Fr^+^, and ^213^Bi^3+^, respectively [72]. In this study, we found that all three cations could be intercalated between the layers of ZrP, but first needed to be converted to the Na‐phase to facilitate rapid kinetics. The ions were held tightly, showing no leaching, in a pH 7.4, 10‐mM carbonate buffer used as an analog for human plasma, which indicated the radionuclides will not be released before reaching the cancerous cells. More recently, we have examined the other alkali metal phases, K‐ZrP, Rb‐ZrP, and Cs‐ZrP, and their subsequent ion‐exchange behavior toward lanthanides and trivalent transition metals [76]. Again, rapid kinetics and tight ion binding were observed, with negligible leaching in a variety of buffer systems.

In this work, we investigate the ion exchange behavior of the previously characterized K‐ and Cs‐ZrP derivatives [76] with ^211^At as the At^+^ and AtO^+^ species, with specific interest in uptake efficiency and binding strength. The ^211^At‐loaded ZrP materials were studied extensively through leaching tests against a variety of buffer solutions. Shielding effects of the materials on the α‐emission were also examined.

## 2 Results and Discussion

### 2.1 Ion Exchange Reactions of A‐ZrP Materials with ^211^At

To begin with, the sorption and uptake of ^211^At on the K‐, and Cs‐derivatives with both the At^+^ and AtO^+^ species were studied. The At^+^ and AtO^+^ were produced by treatment of NaOCl at 40 mM and 1.5 M, respectively. It can be seen in Table 1 that the K‐ZrP showed a strong affinity for both the At^+^ and the AtO^+^ species (≥99% removal), with a preference for the AtO^+^ species. While the Cs‐ZrP material showed lower affinities and the opposite trend, with a preference for At^+^ over AtO^+^. This is believed to be caused by the difference in d‐spacing of the Zr layers and the difference in size of the At^+^ and AtO^+^ cations. The At(I) monovalent cation, At^+^, should be larger than the At(III) monoxide molecular cation, AtO^+^. While neither ion has been directly studied, analogies between the ‐yl ion of AtO^+^ and the actinides can be drawn. For example, the ionic radius of U^3+^ is 1.226 Å [89], while the U radius in the uranyl dioxo cation, UO_2_^+^, is 0.76 Å [90]. The molecular cation is elongated along the At—O bond, but the width perpendicular to the bond should be much smaller, and therefore increase the ease of intercalation between the smaller space of Zr layers of the K‐ZrP material, while the larger At^+^ ion should fit better in the larger space of the Cs‐ZrP layers. It should be noted that the amount of At^+^ or AtO^+^ is much less than the available active sites (<0.1% of the ion exchange capacity), so the materials should remain in the original phase, and the d‐spacing of the layers should not change.

**TABLE 1:** Summary of the uptake of At^+^ and AtO^+^ by the K‐ZrP and Cs‐ZrP materials. Values not in parentheses were calculated from direct measurement of the ^211^At activity in the ZrP materials and the supernate after contact with the ZrP materials, while the values in parentheses were calculated from the ^211^At activity in solution before and after contact with the ZrP materials.

Sample	NaI gamma spectroscopy		Liquid scinitillation counting	
	**K**_**d**_, **mL/g**	%R	**K**_**d**_ **mL/g**	%R
At^+^ K‐ZrP	700 ± 200	99% ± 3%	290 ± 32	97% ± 2%
	(700 ± 30)	(99% ± 2%)	(1,260 ± 140)	(99% ± 1%)
At^+^ Cs‐ZrP	140 ± 10	93% ± 1%	77 ± 1	89% ± 1%
	(140 ± 10)	(94% ± 2%)	(430 ± 8)	(98% ± 1%)
AtO^+^ K‐ZrP	3,000 ± 1,000	100% ± 1%	500 ± 70	98% ± 2%
	(3,000 ± 1,000)	(100% ± 2%)	(2,400 ± 400)	(100% ± 2%)
AtO^+^ Cs‐ZrP	67 ± 3	87% ± 2%	58 ± 2	85% ± 1%
	(70 ± 20)	(87% ± 2%)	(270 ± 10)	(96% ± 2%)

When the uptake of the ^211^At was calculated using LSC, very different values were obtained from measuring the solid directly versus calculating from the solution before and after contact with the ZrP Materials. In the case of measuring the solid directly, there was a consistent decrease in K_d_ by a factor of roughly 0.80. This is in contrast to the same results obtained using the NaI gamma spectrometer, where the values are statistically identical. This is believed to be caused by the shielding of the α‐particles by the dense Zr layers, while the γ‐ and X‐rays are much more penetrating. This will be discussed in detail in a later section.

### 2.2 Leaching of At‐211 from Loaded A‐ZrP Materials

Once loaded, the retention of the ^211^At was examined by contacting the ^211^At‐loaded ZrP materials with a variety of leaching solutions for 24 h. The leaching solutions included a carbonate buffer, a phosphate buffered saline (PBS) buffer, a 4‐(2‐hydroxyethyl)‐1‐piperazineethanesulfonic acid (HEPES) buffer, and solutions of ethylenediaminetetraacetic acid (EDTA) at several concentrations ranging from 0.1 to 10 mM. As can be seen from Table 2, the At^+^ remained tightly bound to both the A‐ZrP materials regardless of what buffer was used to leach the ^211^At, with the exception of the 10 mM EDTA solution, where 1%–2% leaching was observed. The AtO^+^ showed strong binding to the K‐ZrP, except for in the 10 mM EDTA system, where slight leaching was observed. The Cs‐ZrP, on the other hand, showed leaching of 1%–3% in all the buffers except PBS, where no leaching was observed. This is in line with the uptake studies where AtO^+^ showed a much lower affinity towards the Cs‐ZrP. Again, this is believed to be caused by the poor size matching of the slightly smaller AtO^+^ ion and the larger cavity formed by the exchange with the Cs^+^. These results highlight the potential to use the K‐ZrP, and to a lesser extent the Cs‐ZrP, as a delivery mechanism for this chemically challenging radionuclide. It should be noted that future investigations are required to determine biocompatibility and targeting to fully understand the utility of these materials, where the current studies only demonstrate the strong uptake and binding of the radionuclides of interest.

**TABLE 2:** Summary of leaching results with the ^211^At loaded A‐ZrP materials.

Sample	Carbonate	PBS	HEPES	0.1 mM EDTA	1.0 mM EDTA	10 mM EDTA
	pH 7.4	pH 7.4	pH 7.4	pH 7.2	pH 7.3	pH 7.2
At^+^ K‐ZrP	≤0.5%	≤0.5%	≤0.5%	≤0.5%	≤0.5%	2.1% ± 0.2%
At^+^ Cs‐ZrP	≤0.5%	≤0.5%	≤0.5%	≤0.5%	≤0.5%	1.0% ± 0.1%
AtO^+^ K‐ZrP	≤0.5%	≤0.5%	≤0.5%	≤0.5%	≤0.5%	1.4% ± 0.1%
AtO^+^ Cs‐ZrP	2.5% ± 0.3%	≤0.5%	1.0% ± 0.1%	1.0% ± 0.1%	1.0% ± 0.1%	1.6% ± 0.2%

### 2.3 ^211^At α‐Particle Shielding of A‐ZrP Materials

**FIGURE 1:**
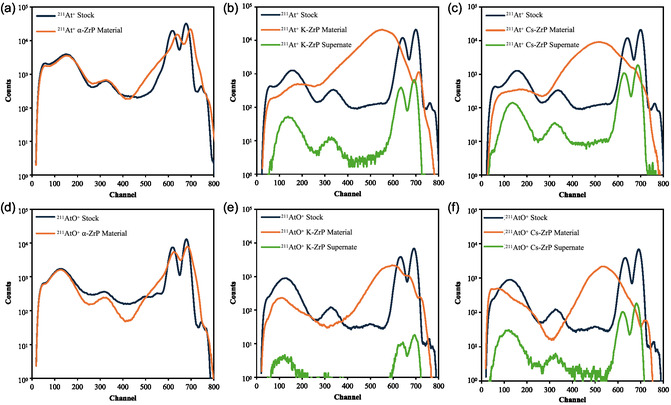
LCS spectra of ^211^At solution before interaction with ZrP material (blue), the ZrP material after interaction with ^211^At (orange), and the ^211^At supernate after interaction with ZrP material (green) for (a) At^+^ with α‐ZrP, (b) At^+^ with K‐ZrP, (c) At^+^ with Cs‐ZrP, (d) AtO^+^ with α‐ZrP, (e) AtO^+^ with K‐ZrP, and (f) AtO^+^ with Cs‐ZrP. Note: only 1/5 of both the ^211^At Stock and ^211^At supernate solutions were used in for LCS measurement, while all of the ^211^At‐loaded ZrP materials were used.

As previously mentioned, the ^211^At emission signal of the γ‐spectroscopy versus the LSC was much different from the ^211^At‐loaded A‐ZrP materials (see Table 1), while for α‐ZrP they were much more similar. This was hypothesized to be caused by a difference in behavior between the tightly bound layers of α‐ZrP, with a d‐spacing of 7.6 Å, and the more loosely bound layers of both K‐ZrP and Cs‐ZrP, where the d‐spacing is 10.7 and 11.6 Å, respectively [76]. In the case of the α‐ZrP, the ^211^At appears to be sorbed to the surface, while in the A‐ZrP materials, it is intercalated between the Zr layers. This is inline with what was observed in our previous work looking at the ^225^Ac decay chain [72]. The Zr layers then shield the α‐particles as they travel through the materials. To test this, the LSC emission spectrum was obtained for each sample. As shown in Figure 1, the spectra from both the stock ^211^At solutions and the supernate after contact with the A‐ZrP materials were nearly identical with respect to the signal energy and shape, where the supernate only had less activity present, producing a less intense signal. There were four major signals. The first two broad signals occurred with a maximum at channels 160 and 340, and are believed to represent the electron‐capture‐decay from either the ^211^At itself or the ^207^Bi daughter. The remaining two signals at channels 650 and 700 are believed to be the α‐emission of ^211^At (5.87 MeV) and ^211^Po (7.59 MeV), respectively. These are much more narrower signals, which are indicative of α‐particles.

**FIGURE 2:**
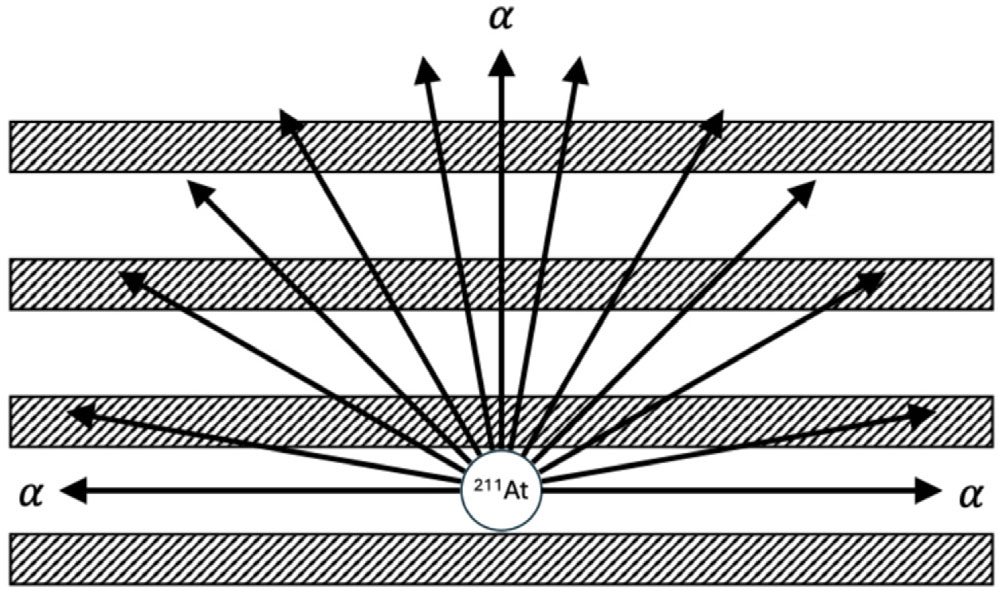
Cartoon of the cross section of the 4π α‐particle emission of ^211^At intercalated between the layers of the ZrP materials.

For α‐ZrP, the signals from the stock ^211^At solutions and those from the α‐ZrP material after interaction with ^211^At are nearly identical, with only a very slight broadening (see Figure 1a,d). This indicates that the ^211^At is mostly on the surface of the particles, so there is minimal interaction of the α‐particles with the α‐ZrP material itself. Conversely, in the case of the A‐ZrP materials, however, there was a significant change in the emission spectrum, where the narrow α‐emissions broaden significantly and shift to lower energies. This is in line with the hypothesis that the ^211^At is intercalated between the Zr layers, and the α‐particles have to traverse through the dense layers. Depending on the angle of emission, the α‐particles, as shown in Figure 2, will travel various lengths through the ZrP materials, losing different amounts of energy based on the trajectory. This will result in α‐particles of different energies interacting with the LCS cocktail, which will produce a broad spectrum of signal energies, and can be observed in Figure 1b,c,e and f). Coincidentally, the lower channel signals were also shifted to lower energy, as well. The shift to lower energy is more pronounced with the Cs‐ZrP materials, with the signal maximum at channel 517 and 533, for At^+^ and AO^+^, respectively, while the maximums for the K‐ZrP materials were 552 and 596, respectively. This, again, is expected, as Cs is much larger than K and should more effectively shield the α‐particles. These results provide clear evidence that the ^211^At is being intercalated in between the layers and that the dense Zr layers are shielding the α‐particles, reducing their energy before they escape from the materials. This shielding effect is advantageous when considering dosimetry concerns upon in vivo administration. This means that the α‐particles will be shielded during circulation and inflict less damage to healthy tissue. Once the ^211^At‐loaded A‐ZrP materials have reached the tumor site, they will still only need to accumulate on the surface of the cell, as the α‐particles should still have enough energy to penetrate 1–2 cell lengths, thereby killing the cell.

## 3 Conclusion

The uptake of ^211^At with the alkali metal phases, K‐ and Cs‐ZrP, has been studied and shown to readily intercalate in between the Zr layers. The K‐ZrP material showed near quantitative uptake, ≥99%, of ^211^At regardless of the speciation, with high affinity for both At^+^ and AtO^+^. The K_d_‐values showed a preference for the At(III) monoxide molecular cation, which is indicative of the smaller metal center fitting into the K^+^ vacancy more efficiently. The Cs‐ZrP material, on the other hand, showed lower overall affinity of both At^+^ and AtO^+^, with an uptake of roughly 94% and 89%, respectively. This indicates that the larger At(I) cation fits more efficiently in the larger Cs^+^ vacancy. The ^211^At LCS spectrum was significantly shifted once it was intercalated between the layers, showing a broad, lower‐energy emission. This is in line with the hypothesis that the α‐emission is occurring primarily inside the particles, rather than on the surface. These results make this class of materials more attractive from a practical standpoint, as the shielding of the α‐particles will reduce the radiation dose to healthy tissue and minimize unwanted side effects. Once intercalated, both At^+^ and AtO^+^ are strongly bound within the K‐ZrP and At^+^ within the Cs‐ZrP and were not released in any of the buffers studied, including a carbonate buffer, a PBS buffer, a HEPES buffer, and solutions of EDTA up to 1 mM.

The efficient uptake, coupled with strong binding of ^211^At, makes K‐ZrP an attractive platform to be further investigated for a delivery vehicle for this TAT radionuclide, with the Cs‐ZrP also showing some potential, with future in vivo studies warranted to demonstrate potential application value. Moreover, the natural shielding of the α‐particles that occurs with both K‐ZrP and Cs‐ZrP provide the added benefit that less dose is delivered to healthy tissue during transportation.

## 4 Experimental

### 4.1 Materials

Nitric acid (ACS Grade, 68%‐70%, HNO_3_), potassium chloride (ACS Grade, 95%–99.5%, KCl), potassium hydroxide (ACS Grade, 100%, KOH), nitric acid (Omnitrace trace metal analysis, 67%–70%, HNO_3_) were purchased from VWR Chemicals BDH; phosphoric acid (ACS Grade, 85%, H_3_PO_4_) was purchased from VWR Chemicals; phosphate‐buffered saline (ACS Grade, PBS) was purchased from MilliporeSigma; cesium hydroxide 50% solution water (Trace metal basis for analysis, 99.9%, CsOH) and zirconyl chloride octahydrate (ACS Grade, 98%, ZrOCl_2_·8H_2_O) were purchased from Acros Organics; cesium chloride (ultrapure, 99.9% metal basis, CsCl) and 4‐(2‐hydroxyethyl)‐1‐piperazineethanesulfonic buffer pH 7.2 (ACS Grade, HEPES) were purchased from Thermo Scientific; ethylenediaminetetraacetic disodium (ACS Grade, EDTA) was purchased from Fisher Chemical company; and sodium bicarbonate (ACS Grade, 7.5% w/v, NaHCO_3_) was purchased from Quality Biological; and all were used as received. Deionized (DI) H_2_O was obtained from an ELGA LabWater Purelab Flex ultrapure laboratory water purification system operated at 18.2 MΩ × cm at 25°C.

### 4.2 Methods

Quantitative analysis for ^211^At was performed via gamma (γ)‐ray spectroscopy using a calibrated ORTEC GEM Coaxial P‐type HPGe Gamma‐Ray Detector (HPGe) with a relative efficiency of 20.0% and an active detector volume of ˜ 115 cm^3^ and DSPEC‐50 digital signal analyzer along with MAESTRO Software. The detector has an energy resolution of 0.82 at 122 keV and 1.33 at 1330 keV. Relevant nuclear data were obtained from Browne and Firestone [91]. All calibrations were determined with a ^152^Eu and a ^207^Bi standard γ‐ray source, both traceable to the National Institute of Standards and Technology (NIST) purchased from Eckert & Ziegler Isotope Products. The ^211^At was tracked directly using the 76.9, 79.3, 89.8, and 92.3 keV X‐rays and 687 keV γ‐ray. In addition to the HPGe, a PerkinElmer 2470 WIZARD^2^ Automatic Gamma Counter with well‐type NaI detectors was employed to perform semi‐quantitative analysis to compare total ^211^At count rates in the aqueous and solid phases for the ion exchange experiments. As well as, a liquid scintillation counting (LSC) on a HIDEX 300 SL Automatic Liquid Scintillation Counter using Safety‐Solve Complete Counting Cocktail, purchased from Research Products International. The data were obtained using MikroWin 2000 software. All experiments were performed in triplicate. **WARNING**: ^211^At is highly radioactive and was handled under ALARA principles in laboratories equipped to handle radioactive materials appropriately.

### 4.3 Shipment and Recovery of At‐211 from 3‐Octanone Impregnated Columns

The shipment and recovery of the ^211^At‐impregnated column have previously been reported in detail, but will be summarized here. The separation columns, made in‐house at Texas A&M University, were comprised of 0.5 mL of 3‐octanone impregnated Amberchrom CG300M porous beads with a pore volume of 0.7 mL g^−1^ and a particle size of 50–100 µm packed into a 1‐mL Thermo Scientific Pierce Spin Column [41, 43, 45]. Once loaded with ^211^At, they were shipped from College Station, TX, to Birmingham, AL, where the ^211^At was recovered from the column by elution with 3‐octanone [47]. The ^211^At was then recovered in either the At^+^ or the AtO^+^ species by reaction with OCl^–^. This was accomplished by contacting the ^211^At‐containing 3‐octanone with 0.04 or 1.5 M NaOCl solution, respectively.

### 4.4 Synthesis of Potassium and Cesium Derivatives of Alpha‐Zirconium Phosphate

The synthesis and characterization of the alpha‐zirconium phosphate (α‐ZrP) and modification to the K‐ and Cs‐phases have been previously reported in detail, with a summary provided here, and additional details found in the Supporting Information [76]. The synthesis of α‐ZrP was as follows: a roughly 0.8 M ZrOCl_2_·8H_2_O solution dissolved in H_2_O was placed in the PPL liner of a Tosyuwir 100 mL hydrothermal synthesis autoclave reactor. To ensure homogeneity, the solution was stirred continuously while 4 M H_3_PO_4_ was added dropwise. The final solution composition was two parts 0.8 M Zr solution and 3 parts 4 M H_3_PO_4_, with the total volume kept at less than 50% of the reactor volume. A white precipitate formed upon the addition of the H_3_PO_4_. The mixture was then sealed in the reactor and placed in a VWR forced air oven at 200°C for 6 h, and then allowed to cool on the benchtop overnight. Once cooled, the white solid was washed copiously with H_2_O via centrifugation and decantation to remove any excess H_3_PO_4_. The powder was dried at 30°C for 24 h. The resulting white solid was then ground to a fine powder with a mortar and pestle. Conversion to the K‐phase and Cs‐phase was then achieved by reaction with KOH‐KCl and CsOH‐CsCl solution, respectively, until the equilibrium pH of the supernate reached 10.2. The A‐ZrP materials were then washed and dried in the same manner as the α‐ZrP.

### 4.5 Ion Exchange Reactions with Potassium and Cesium Derivatives of Alpha‐Zirconium Phosphate

Batch ion exchange studies were conducted by contacting roughly 50 mg of the A‐ZrP material with 0.5 mL solution of At^+^ or AtO^+^ for a minimum of 10 min, where the ^211^At activity was 150 nCi and 200 nCi, respectively. The mixture was vigorously mixed, followed by end‐over‐end tumbling on a VWR tube rotator at roughly 18 rpm for a minimum of 10 min. The solid and liquid phases were separated by centrifugation and decantation. A 100‐µL aliquot of the supernate was mixed with 1 mL of safety‐solve complete counting cocktail, while 1 mL of the cocktail was added directly to the ^211^At‐loaded A‐ZrP materials. Both phases were analyzed by gamma counting and LSC. The ion exchange was measured by calculating the distribution coefficient (Equation (1)), *K*_*d*_, and the percent removal (Equation (2)), R%,

(1) $$K_{d}=\frac{A_{\mathrm{aq}\;I}-A_{\mathrm{aq}\;\mathrm{Eq}}}{A_{\mathrm{aq}\;\mathrm{Eq}}}\cdot \frac{V}{m}=\frac{A_{s\;\mathrm{Eq}}}{A_{\mathrm{aq}\;\mathrm{Eq}}}\cdot \frac{V}{m}$$

where *A*_aq_ and *A*_s_ are the activities of ^211^At in solution and the loaded A‐ZrP materials, respectively, *V* is the volume of metal solution in mL, and *m* is the mass of the exchanger in g.

(2) $$R\%=\frac{A_{\mathrm{aq}\;I}-A_{\mathrm{aq}\;\mathrm{Eq}}}{A_{\mathrm{aq}\;\mathrm{Eq}}}\times 100\%=\frac{A_{s\;\mathrm{Eq}}}{A_{\mathrm{aq}\;\mathrm{Eq}}}\times 100\%$$

### 4.6 Leaching of the ^211^At‐Loaded Potassium and Cesium Derivatives Alpha‐Zirconium Phosphate Phases

Batch leaching experiments of the ^211^At‐loaded A‐ZrP materials were conducted by taking roughly 50 mg of the A‐ZrP material and contacting it with 500 µL of a 150 nCi and 200 nCi ^211^At solution for At^+^ and AtO^+^, respectively. The mixture was vigorously mixed on a vortexer for several seconds, followed by end‐over‐end tumbling at roughly 18 rpm for a minimum of 10 min. The solid and liquid phases were separated by centrifugation and decantation, and both phases were analyzed by gamma counting. The ^211^At‐loaded A‐ZrP material was then interacted with a 1‐mL leaching solution (see Table 3), and the two phases were again mixed vigorously with a vortexer for several seconds, followed by end‐over‐end tumbling at roughly 18 rpm for 24 h. The samples were then centrifuged, and the liquid and solid phases were separated by decantation. A 100–µL aliquot of the supernate was sampled and analyzed by gamma counting and LSC. The leaching was measured by calculating the percent leached (Equation (3)), L%,

**TABLE 3:** Details of leaching solutions.

Buffer	Concentration	pH
Carbonate	8.9 mM	7.4
PBS	4.0 mM	7.4
HEPES	10 mM	7.4
10 mM EDTA	10 mM	7.2
1.0 mM EDTA	1.0 mM	7.3
0.1 mM EDTA	0.10 mM	7.2

(3) $$L\%=\frac{A_{aq\;Eq}}{A_{s\;Eq}+A_{aq\;Eq}}$$

where *A*_aq_ and *A*_s_ are the activities of ^211^At in solution and the loaded A‐ZrP materials, respectively.

## Supporting Information

Additional supporting information can be found online in the Supporting Information section.

## Author Contributions

**B. D. Imansha Madhushan**: conceptualization, data curation, formal analysis, methodology, validation, writing – original draft. **Adrianna L. Orsi**: data curation, formal analysis. **Jehan S. Perera**: conceptualization, data curation, formal analysis, methodology, validation, writing – review and editing. **Jennifer M. Pyles**: data curation, formal analysis, methodology, validation, writing – review and editing. **Christine C. Lawrence**: data curation. **Marcus Le**: data curation. **Gabriel C. Tabacaru**: – funding acquisition, project administration, supervision, writing – review and editing. **Lauren A. McIntosh**: – funding acquisition, project administration, supervision, writing – review and editing. **Sherry J. Yennello**: funding acquisition, project administration, supervision, writing – review and editing. **Jonathan D. Burns**: conceptualization, funding acquisition, methodology, project administration, supervision, writing – original draft.

## Funding

This work was supported by the Office of Isotope R and D and Production (DE‐SC0024600, DE‐SC0022550 and DE‐SC0020958) and Nuclear Physics (DE‐FG02‐93ER40773).

## Conflicts of Interest

The authors declare the following financial interests/personal relationships, which may be considered as potential competing interests: Jonathan D. Burns, Lauren A. McIntosh, Gabriel C. Tabacaru, and Sherry J. Yennello have patent #PCT/US21/25156 “Rapid At‐211 purification method” and Jonathan D. Burns, Lauren A. McIntosh, Gabriel C. Tabacaru, and Sherry J. Yennello have patent # PCT/US21/63241 “Systems and Methods for Automated Separation and Recovery of Astatine” pending to Texas A&M University.

## Supporting information

Supplementary Material

## Data Availability

The datasets generated during and/or analyzed during the current study are available from the corresponding author on reasonable request.
