# Supplementary material for: Uptake and Binding of At‐211 Into K‐ and Cs‐Derivatives of Alpha‐Zirconium Phosphate Nanoplatelets for Use as a Targeted Alpha Therapy Delivery Platform
Source: Small Sci. 2026 Apr 18;6(4):e202500640. doi: 10.1002/smsc.202500640 (PMC13091646; doi:10.1002/smsc.202500640)
Supplement: Supplementary file 1 — Supplementary Material [file SMSC-6-e202500640-s001.pdf]

Supporting Information

Uptake and Binding of At-211 into K- and Cs- Derivatives of alpha-Zirconium Phosphate Nanoplatelets for use as a Targeted Alpha Therapy Delivery Platform

B. D. Imansha Madhushan[1], Adrianna L. Orsi[1], Jehan S. Perera[1], Jennifer M. Pyles[1], Christine C. Lawrence[2], Marcus Le[2,3], Gabriel C. Tabacaru[2], Shayden R. Fritz[2,3], Lauren A. McIntosh[2], Sherry J. Yennello[2,3], and Jonathan D. Burns[*,1]

[1]Department of Chemistry, University of Alabama at Birmingham, Birmingham, AL 35294, USA
[2]Cyclotron Institute, Texas A&M University, College Station, TX 77843, USA
[3]Department of Chemistry, Texas A&M University, College Station, TX 77843, USA

For submission to publish in the
Small Science
as a full-length article.

This submitted report has been authored by the University of Alabama at Birmingham, under Award No. DE-SC0024600 with the U.S. Department of Energy. The United States Government retains and the publisher, by accepting the article for publication, acknowledges that the United States Government retains a non-exclusive, paid-up, irrevocable, world-wide license to publish or reproduce the published form of this manuscript, or allow others to do so, for the United States Government purposes. *The Department of Energy will provide public access to these results of federally sponsored research in accordance with the DOE Public Access Plan (http://energy.gov/downloads/doe-public-access-plan).*

This report was prepared as an account of work sponsored by an agency of the United States Government. Neither the United States Government nor any agency thereof, nor any of their employees, makes any warranty, express or implied, or assumes any legal liability or responsibility for the accuracy, completeness, or usefulness of any information, apparatus, product, or process disclosed, or represents that its use would not infringe privately owned rights. Reference herein to any specific commercial product, process, or service by trade name, trademark, manufacturer, or otherwise does not necessarily constitute or imply its endorsement, recommendation, or favoring by the United States Government or any agency thereof. The views and opinions of authors expressed herein do not necessarily state or reflect those of the United States Government or any agency thereof.

Research supported by U.S. Department of Energy Office of Isotope R&D and Production and the DOE Established Program to Stimulate Competitive Research (EPSCoR) under Award No. DE-SC0024600, ALO acknowledges financial support from the U.S. Department of Energy, Office of Science, Isotope Program, under Award Number DE-SC0022550 through the Horizon-broadening Isotope Production Pipeline Opportunities (HIPPO) program. CCL, ML, GCT, SRF, LAM, and SJY acknowledge financial support from U.S. Department of Energy Isotope Program, managed by the Office of Science for Isotope R&D and Production under Award No. DE-SC0020958, and Texas A&M University through the Bright Chair in Nuclear Science. Additionally, this work was enabled by the Texas A&M Nuclear Solutions Institute and U.S. Department of Energy under Award No. DE-FG02-93ER40773.

Supporting Information

Uptake and Binding of At-211 into K- and Cs- Derivatives of alpha-Zirconium Phosphate Nanoplatelets for use as a Targeted Alpha Therapy Delivery Platform

B. D. Imansha Madhushan[1], Adrianna L. Orsi[1], Jehan S. Perera[1], Jennifer M. Pyles[1], Christine C. Lawrence[2], Marcus Le[2,3], Gabriel C. Tabacaru[2], Shayden R. Fritz[2,3], Lauren A. McIntosh[2], Sherry J. Yennello[2,3], and Jonathan D. Burns[*,1]

[1]Department of Chemistry, University of Alabama at Birmingham, Birmingham, AL 35294, USA
[2]Cyclotron Institute, Texas A&M University, College Station, TX 77843, USA
[3]Department of Chemistry, Texas A&M University, College Station, TX 77843, USA

*e-mail: burnsjon@uab.edu

Experimental

Materials:

Nitric acid (ACS Grade, 68-70%, HNO_3), potassium chloride (ACS Grade, 95-99.5%, KCl), potassium hydroxide (ACS Grade, 100%, KOH), nitric acid (Omnitrace® trace metal analysis, 67-70%, HNO_3) were purchased from VWR Chemicals BDH; phosphoric acid (ACS Grade, 85%, H_3PO_4), chromium(III) nitrate nonahydrate (ACS grade, 98.5%, $Cr(NO_3)_3 \bullet 9H_2O$), and tampon phosphate pH 7.2 buffer (ACS Grade) were purchased from VWR Chemicals; cesium hydroxide 50% solution water (Trace metal basis for analysis, 99.9%, CsOH) and zirconyl chloride octahydrate (ACS Grade, 98%, $ZrOCl_2 \cdot 8H_2O$) were purchased from Acros Organics; rubidium hydroxide 50% w/w aqueous solution (meta basis, 99.6%, RbOH), rubidium chloride (trace metal basis, 99.8+%, RbCl), cesium chloride (ultrapure, 99.9% metal basis, CsCl), and 4-(2-hydroxyethyl)-1-piperazineethanesulfonic buffer pH 7.2 (ACS Grade, HEPES) were purchased from Thermo Scientific; ethylenediaminetetraacetic disodium(ACS Grade, EDTA) was purchased from Fisher Chemical company; sodium bicarbonate (ACS Grade,7.5% w/v, $NaHCO_3$) was purchased from Quality Biological; lanthanum chloride heptahydrate (ACS Grade, $LaCl_3 \bullet 7H_2O$) was purchased from Sigma Chemical company; and all were used as received. Deionized (DI) H_2O was obtained from an ELGA LabWater Purelab Flex ultrapure laboratory water purification system operated at 18.2 MΩ×cm at 25°C.

Methods:

X-ray powder diffraction (XRPD) patterns were collected on a Panalytical Empyrean X-ray diffractometer with copper X-ray source (λ = 1.5406 Å), carried out at 45 kV and 40 mA, with a scan range of 5–45° 2θ by the reflection-transmission spinner method (step 0.013°, time 0.1 s). Thermogravimetry analysis (TGA) was performed using a PerkinElmer Thermogravimetric Analyzer TGA 8000 at a heating rate of 5 °C per min^{-1} up to 800 °C under N_2 airflow. Attenuated total reflectance Fourier transform infrared (ATR-FT-IR) analyses were done by using a PerkinElmer Spectrum Two™ FT-IR Spectrometer and Analysis Systems equipped with

a PIKE Technologies Inc. MIRacle^{TM} ATR diamond crystal plate single reflection accessory from 500–4000 cm^{-1}, averaging 16 scans per spectrum. Scanning electron microscopy (SEM) measurements were performed on a FEI Quanta 650 FEG scanning electron microscope. The SEM electron beam was operated at an accelerating voltage of 5 kV, and the images were recorded from the secondary electron (SE) detector signal. SEM data was collected using XT microscope control software. Samples were sputtered using Denton Desk V with a platinum/palladium target. Transmission electron microscopy (TEM) analysis was performed using a JEOL 1400 flash electron-Ins microscope. The beam energy for the electron was 120 kV. An AMT-NanoSprint43L-MarkII Camera was used to take the image. X-ray photoelectron spectroscopy (XPS) was performed using the Phi Electronics VersaProbe 5000 spectrometer, featuring a micro-focused AI monochromatic source (λ = 1486.6 eV) and a dual anode conventional X-ray source equipped with a neutralizer. Survey spectra were captured with a step size of 0.8 eV and pass energy of 187.85 eV, while high-resolution scans had a step size of 0.1 eV with a pass energy of 23.5 eV.

Synthesis of alpha-Zirconium Phosphate:

Alpha-zirconium phosphate (α-ZrP) was synthesized on the 5-g scale under hydrothermal conditions using a Tosyuwir 100 mL hydrothermal synthesis autoclave reactor equipped with polypropylene (PPL) liner. The general procedure was to dissolve 5.336 g of $ZrOCl_2{\bullet}8H_2O$ in 20 mL of H_2O in the PPL liner. While stirring, 30 mL of 4 M H_3PO_4 was added dropwise. The final ratio of P:Zr was roughly 7.25:1. Once mixed, the PPL liner was sealed in the hydrothermal synthesis autoclave reactor and heated at 200 ºC for 6 h in a VWR forced air oven. The reactor was then removed from the oven and allowed to cool overnight. The product was then washed with H_2O followed by centrifugation and decantation of the supernate. Several washes were conducted to remove the excess H_3PO_4. The powder was dried at 30 ºC for 24 h. The resulting white solid was then ground to a fine powder with a mortar and pestle.

Characterization of A-ZrP Materials:

The synthesis and characterization have already been discussed previously [1] in detail, but are summarized here for clarity of the current work.

As shown in Figure S1, XRPD analyses confirm that the larger cations were being intercalated between the layers (α-ZrP) (see Table S1).

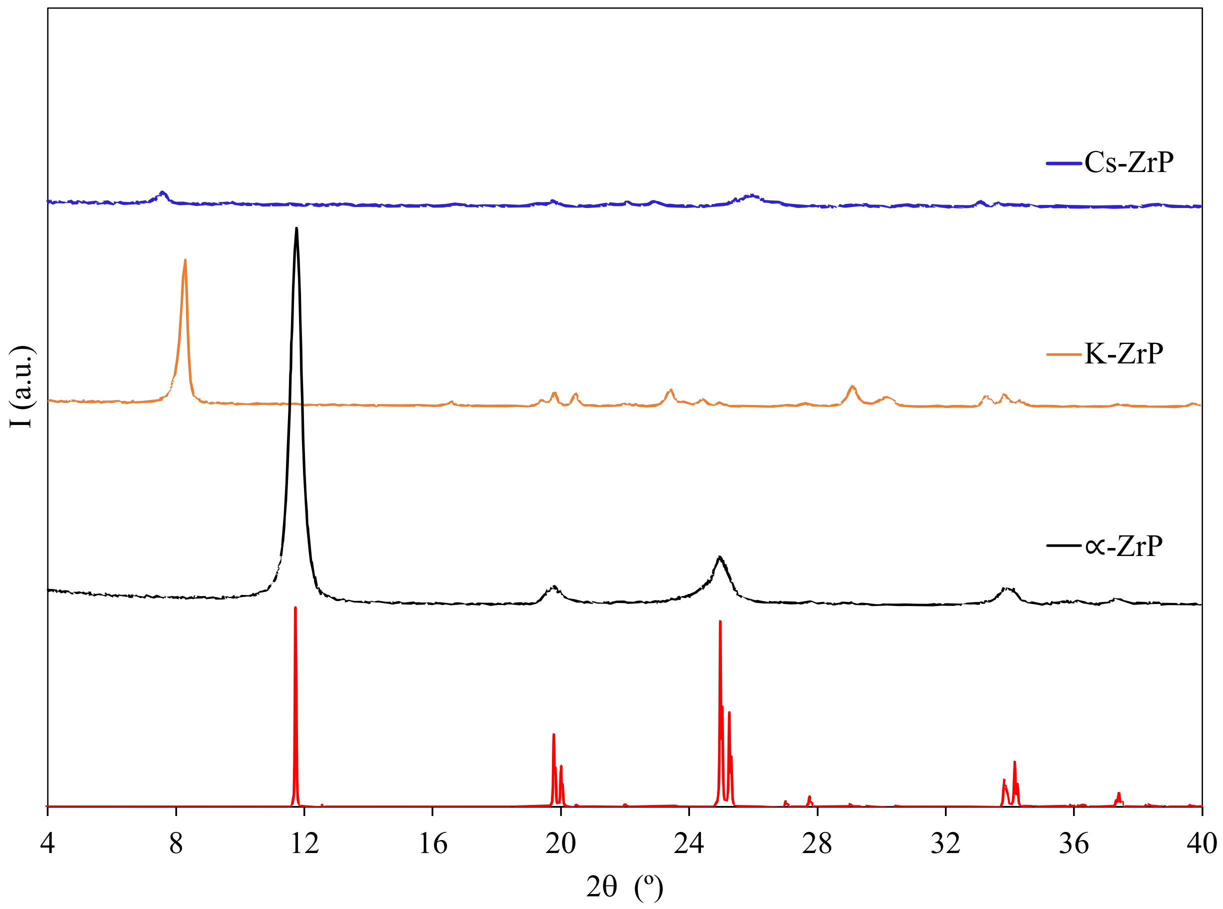

Figure S1: Powder X-ray diffraction patterns of the pristine α-ZrP and the K-ZrP and Cs-ZrP phases, as adapted from Ref [1]. The red lines indicate the calculated pattern from the crystal structure of $Zr(O_3POH)_2 \cdot H_2O$.[2]

Table S1: Observed d-spacing for the 002 reflection planes of the pristine α-ZrP and the hydrated and dry K-ZrP, Rb-ZrP, and Cs-ZrP phases, as adapted from Ref [1].

Sample	2θ (002)	d-spacing (Å)
α-ZrP	11.75	7.5
K-ZrP	8.28	10.7
Cs-ZrP	7.64	11.6

The water content of the materials was determined by TGA measurements, and the weight loss curves are shown in Figure S2. The percent water content has calculated and summarized in Table S2.

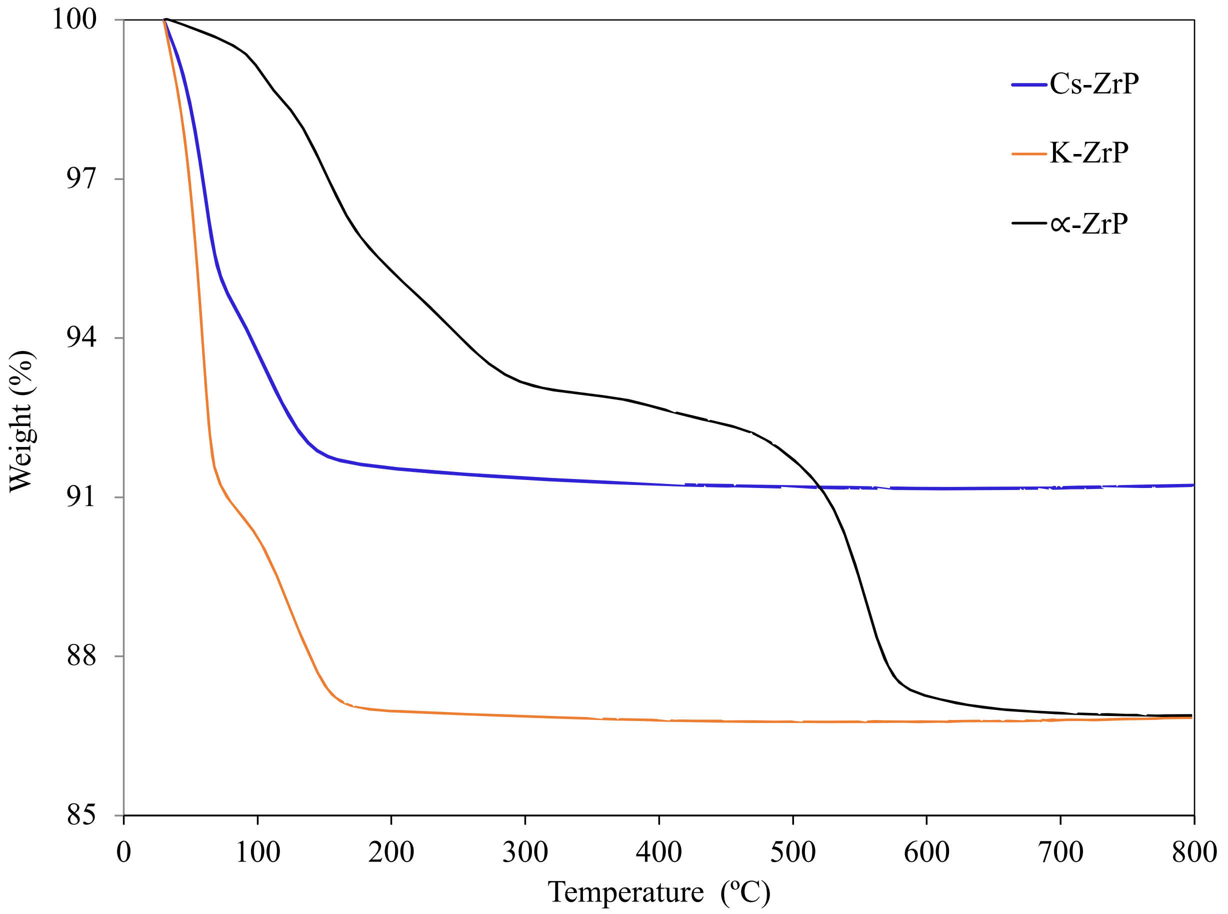

Figure S2: Thermogravimetric analysis of the pristine α-ZrP and the K-ZrP and Cs-ZrP phases, as adapted from Ref [1]..

Table S2: Summary of the TGA weight loss for K-ZrP and Cs-ZrP phases, as adapted from Ref [1], for the surface water (0–100 ºC) and the bound water (100-200 ºC).

Sample	0–100 ºC	100–200 ºC	Total
K-ZrP	9.77%	3.25%	13.15%
Cs-ZrP	6.29%	2.16%	8.78%

The chemical formulas and estimated formal weight of the materials are summarized in Table S3. The materials have roughly 3 mols of H_2O per unit formula.

Table S3: Summary of the characterization and analysis of the A-ZrP materials, as adapted from Ref [1].

Sample	Formula	*n*	FW
K-ZrP	$Zr(O_3POK)_2 \bullet nH_2O$	3.2	416
Cs-ZrP	$Zr(O_3POH)_{0.1}(O_3POCs)_{1.9} \bullet nH_2O$	2.9	587

The ATR-FT-IR spectra were obtained and are shown in Figure S3.

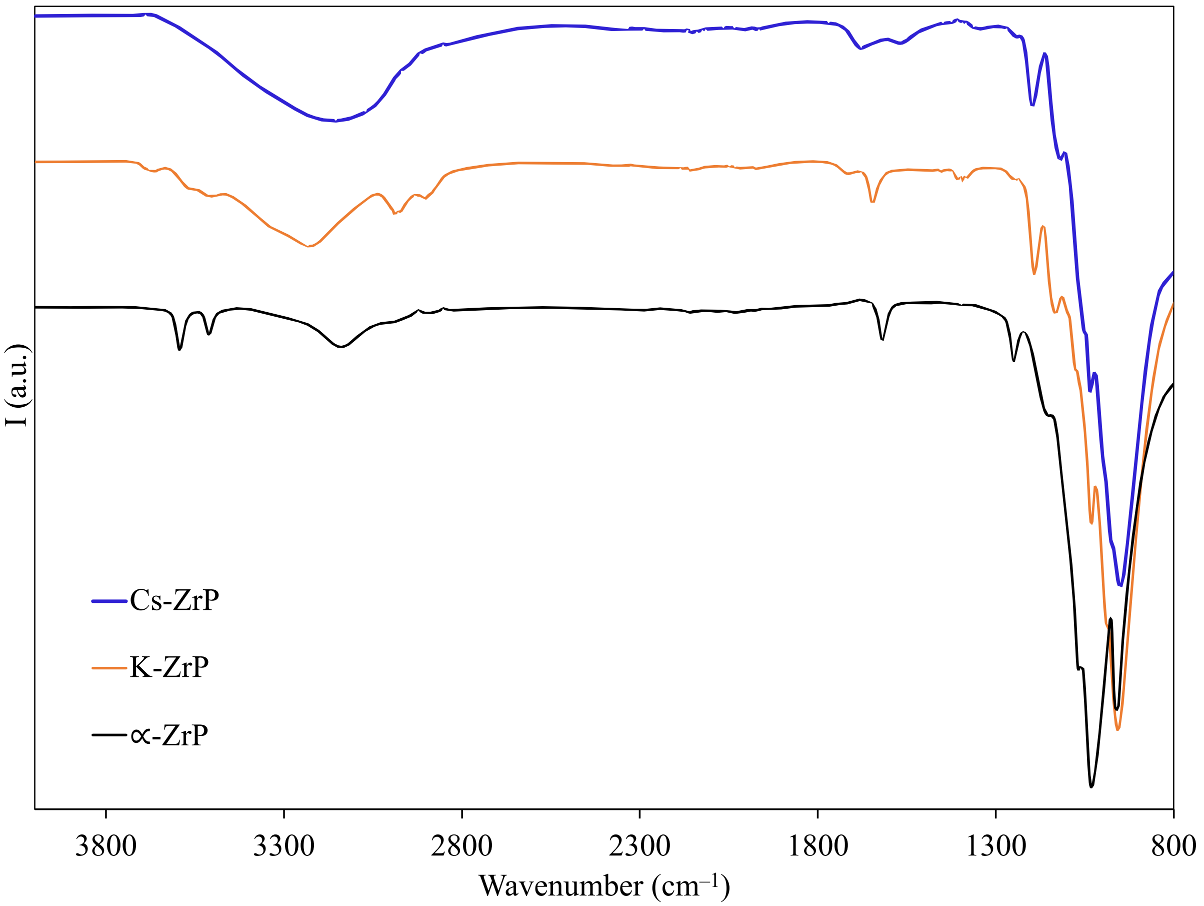

Figure S3: Attenuated total reflectance Fourier transform infrared analysis of the pristine α-ZrP and the K-ZrP and Cs-ZrP phases, as adapted from Ref [1].

The SEM and TEM images of the materials are shown in Figure S4.

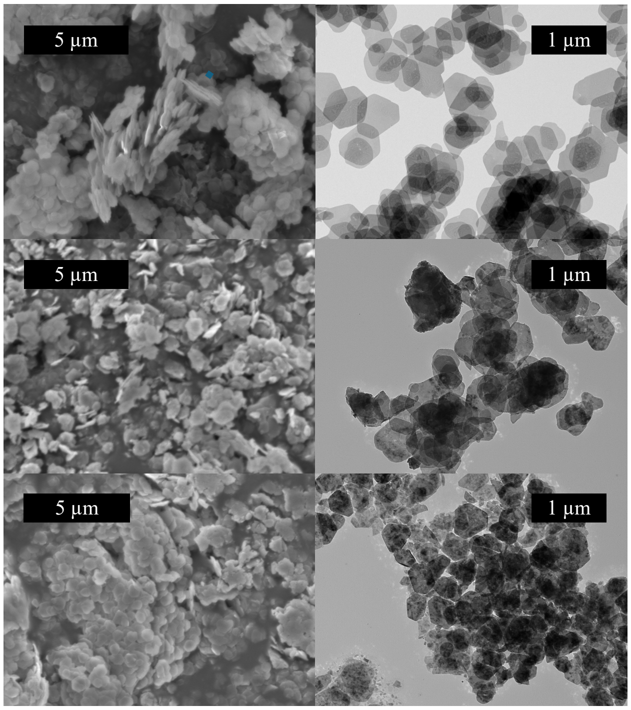

Figure S4: SEM (left column) and TEM (right column) of the pristine α-ZrP (top row), the K-ZrP (middle row), and Cs-ZrP (bottom row) phases, as adapted from Ref [1].

The XPS analysis confirming no changes in structure occur other than the exchange of H^+ for K^+ or Cs^+ and the subsequent expansion of the layers, as can be seen in Figure S5. The P:Zr ratio remained approximately 2:1 in all cases (see Table S4).

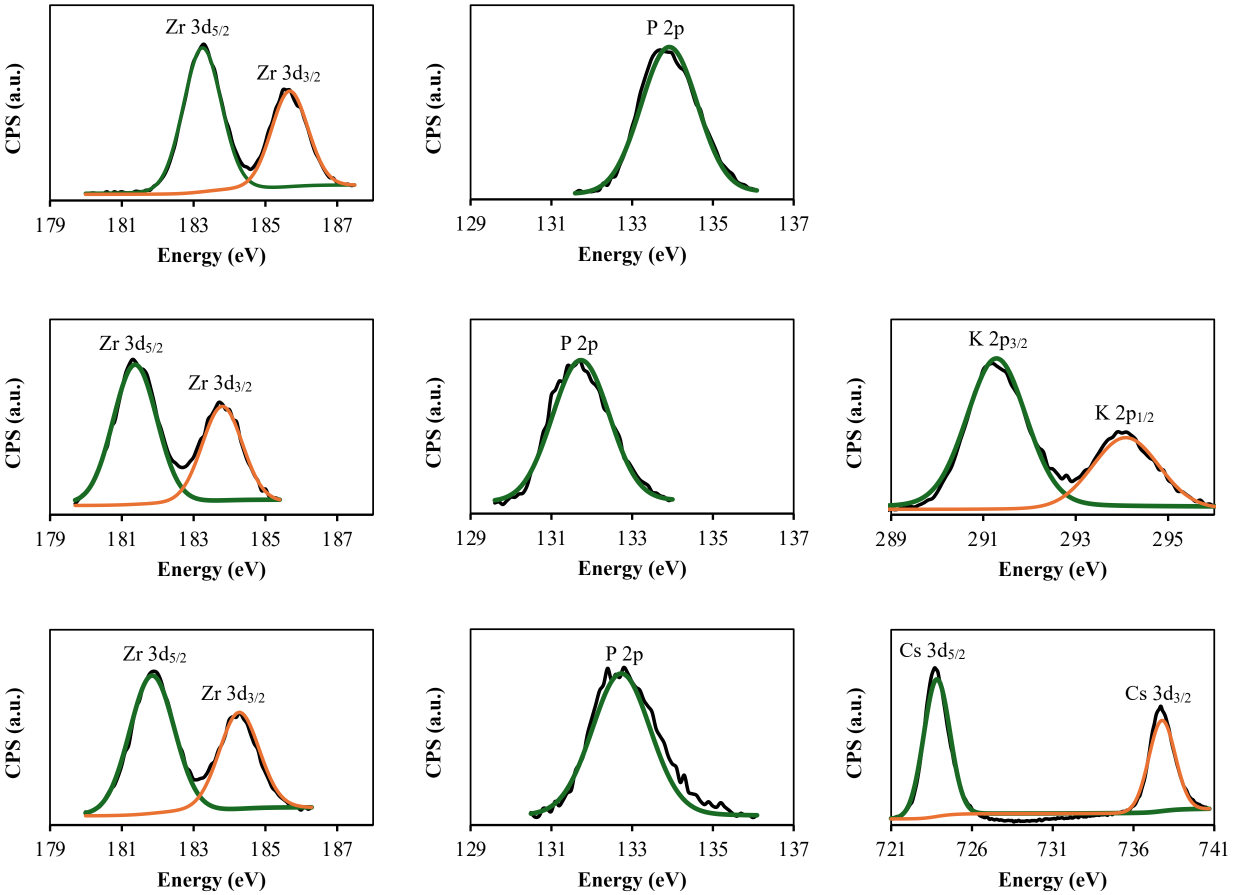

Figure S5: XPS analysis of the pristine α-ZrP (top row), the K-ZrP (middle row), and the Cs-ZrP (bottom row) phases, as adapted from Ref [1].

Table S4: Summary of the XPS characterization for P and Zr in the A-ZrP materials, as adapted from Ref [1].

Sample	P (%)	Zr (%)	P:Zr
α-ZrP	15.8	7.2	2.2
K-ZrP	13.8	6.9	2.0
Cs-ZrP	13.9	7.3	1.9

References:

1. Imansha Madhushan, B. D.; Orsi, A. L.; Pyles, J. M.; Burns, J. D., *Dalton Trans.* **2025,** *54* (44), 16344-16355. DOI https://doi.org/10.1039/D5DT02047F.

2. Troup, J. M.; Clearfield, A., *Inorg. Chem.* **1977,** *16* (12), 3311-3314. DOI https://doi.org/10.1021/ic50178a065.
